# Aberrant expression of minichromosome maintenance protein-2 and Ki67 in laryngeal squamous epithelial lesions

**DOI:** 10.1038/sj.bjc.6601234

**Published:** 2003-09-09

**Authors:** P Chatrath, I S Scott, L S Morris, R J Davies, S M Rushbrook, K Bird, S L Vowler, J W Grant, I T Saeed, D Howard, R A Laskey, N Coleman

**Affiliations:** 1 MRC Cancer Cell Unit, Hutchison/MRC Research Centre, Hills Road, Cambridge CB2 2XZ, UK; 2 Royal National Throat Nose and Ear Hospital, 330 Grays Inn Road, London WC1X 8DA, UK; 3 Centre for Applied Medical Statistics, University Forvie Site, Robinson Way, Cambridge CB2 2SR, UK; 4 Department of Histopathology, Addenbrooke's Hospital, Hills Road, Cambridge CB2 2QQ, UK; 5 Department of Histopathology, Harold Wood Hospital, Gubbins Lane, Romford, Essex RM3 0BE, UK

**Keywords:** larynx, squamous cell carcinoma, squamous dysplasia, early diagnosis, minichromosome maintenance protein-2, laryngeal brushing

## Abstract

Histological classification of laryngeal epithelial lesions is highly subjective, and methods of cytological detection are not well developed. Improved determination of aberrant cell cycle entry may allow increased objectivity in histological assessment and enable the development of less invasive diagnostic cytology tests. Sections of normal larynx (*n*=10), laryngeal dysplasia (*n*=20) and laryngeal squamous cell carcinoma (SCC) (*n*=10) were classified according to the Ljubljana classification and stained for markers of cell cycle entry, minichromosome maintenance protein-2 (Mcm-2) and Ki67. Expression patterns were compared using double labelling confocal microscopy. There was a correlation between Mcm-2 and Ki67 labelling indices (*ρ*=0.93; 95% CI [0.84, 0.97]) and both markers showed increased expression from normal epithelium to SCC (Mcm-2, *P*=0.001; Ki67, *P*=0.0002). Importantly, there was minimal expression of Mcm-2 or Ki67 in the most superficial layers of normal larynx and abnormal or atypical hyperplasia, in contrast to carcinoma *in situ* and SCC. Clusters of Mcm-2/5-positive cells were present in cytological preparations from SCC, but not from those showing atypical hyperplasia or inflammation in non-neoplastic tissue. Minichromosome maintenance protein-2 staining may increase the objectivity and reliability of histological grading of laryngeal epithelial lesions. Laryngeal brushings, combined with immuno-enhanced liquid-based cytology, could be useful, as a less invasive approach, to the detection of laryngeal malignant and premalignant lesions.

Along with carcinoma of the oral cavity, laryngeal squamous cell carcinoma (SCC) is the most common primary tumour of the head and neck, excluding the skin (Watkinson *et al*, 2000; Weiss and Goldblum, 2001). It progresses through several premalignant or dysplastic stages in a manner broadly similar to that of cervical SCC (Hellquist *et al*, 1999; Gale *et al*, 2000; Weiss and Goldblum, 2001). Diagnosis and differentiation of the different grades of dysplastic laryngeal lesions has traditionally been accomplished by histological analysis, although it is widely acknowledged that currently available criteria give rise to an inevitable degree of subjectivity. Such histological variability is not helped by the fact that precancerous lesions of the larynx have no specific laryngoscopic appearances and can be variously interpreted as chronic laryngitis, leukoplakia, keratosis or erythroplakia (Gale *et al*, 2000; Weiss and Goldblum, 2001). It is important, therefore, to improve the criteria for histological diagnosis of different grades of dysplasia and to be able more reliably to differentiate those lesions likely to progress to invasive malignancy from those lesions likely to regress or respond to conservative treatment.

Laryngeal dysplasia (LD) has traditionally been classified in a similar fashion to premalignant lesions of the cervix (Kambic and Gale, 1995; Kambic, 1997). In the WHO classification, for example, a minor degree of atypical change confined to the basal third of the squamous epithelium is defined as laryngeal dysplasia grade 1 (LD-1). In LD-2, there is involvement of the basal one-third to two-thirds of the epithelium and LD-3 includes carcinoma *in situ* and severe dysplasia, with or without surface keratosis, in which atypia involves at least the basal two-thirds of the epithelium (Michaels, 1992).

There are major aetiological differences between carcinomas of the larynx and the cervix, particularly in respect of the importance of human papilloma virus (HPV). In contrast to its role in the cervix, HPV appears to be of relatively minor significance in the generation of LD (Kambic, 1977; Smith *et al*, 2000). The Ljubljana classification of LD (Hellquist *et al*, 1999) takes into account the differences in the aetiology and pathology of laryngeal compared to cervical lesions. The histological system comprises four grades. Simple hyperplasia and abnormal hyperplasia (corresponding to simple squamous cell hyperplasia in the WHO scheme) are regarded as benign lesions. Atypical hyperplasia (WHO: LD-1 and LD-2) is a lesion with some risk of development of invasive carcinoma. Carcinoma *in situ* (WHO: LD-3 and carcinoma *in situ*) is a lesion with a high risk of development of invasive carcinoma (Hellquist *et al*, 1999). These subdivisions broadly reflect those of other classification systems, although it is felt that the Ljubljana method more reliably predicts those lesions that are likely to progress to invasive carcinoma.

Programmes for the early detection of premalignant and malignant disease of the larynx are not yet well developed, largely because of the difficulties inherent in examining this organ. Examination has to be performed under general anaesthesia and the small, but not insignificant risk involved in taking biopsies prevents this from occurring unless the clinical index of suspicion is high. To further complicate matters, premalignant conditions do not show gross diagnostic appearances, which can be identified under direct endoscopy (Kambic, 1997; Watkinson *et al*, 2000). Less invasive diagnostic tests are required if safety, affordability and patient compliance rates are to improve.

Previous reports have suggested that laryngeal brushings are of limited value for early diagnosis, because of a poor yield of surface epithelial cells, contamination with blood necrotic debris and surface keratosis (Vowles *et al*, 1997). However, these studies were performed before modern cytological techniques were widely available. With the application of liquid-based cytological techniques, coupled with novel biomarkers used to detect neoplastic cells (Biscotti *et al*, 2002; Davies *et al*, 2002), laryngeal brushing may now represent a viable less-invasive technique for the detection of neoplasia in the larynx, as has been shown to be the case in cervix and colon (Williams *et al*, 1998; Davies *et al*, 2002).

Promising biomarkers for improving cancer detection include the minichromosome maintenance (MCM) proteins (MCMs 2–7), which assemble in the prereplication complex and are essential for DNA replication in eukaryotic cells (Kearsey and Labib, 1998). All six proteins are abundant throughout the cell cycle and are broken down rapidly on differentiation and more slowly in quiescence (Maiorano *et al*, 1996; Kearsey and Labib, 1998; Musahl *et al*, 1998; Tanaka and Diffley, 2002). Antibodies against MCMs detect more cells in tissues than other ‘proliferation’ markers such as Ki67 (Todorov *et al*, 1998; Williams *et al*, 1998; Freeman *et al*, 1999). We and others have shown that immunocytochemical staining for MCMs such as Mcm-2 or Mcm-5 may be of value in identifying malignant or premalignant lesions in a range of clinical specimens (Todorov *et al*, 1998; Williams *et al*, 1998; Freeman *et al*, 1999; Stoeber *et al*, 1999; Meng *et al*, 2001; Davies *et al*, 2002; Going *et al*, 2002; Hunt *et al*, 2002; Rodins *et al*, 2002).

In the present study, we have investigated different grades of LD for expression of markers of cell cycle entry (Mcm-2 and Ki67) and have used double-labelling confocal microscopy to compare the expression of Mcm-2 and Ki67 in individual specimens. We have compared levels of expression of both markers in LD with those seen in normal epithelium and laryngeal SCC. In particular, we have assessed the distribution of staining in the superficial third of the laryngeal epithelium, to determine whether or not there is any potential for exploiting aberrant surface expression of Mcm-2 to detect precancerous lesions cytologically by brushing the epithelium at laryngoscopy. Based on our findings, we have undertaken a small pilot study to examine whether immunocytochemical analysis of MCM expression in liquid-based cytology specimens from laryngeal brushings may represent a sensitive and novel approach to detection of malignant and potentially premalignant laryngeal lesions.

## MATERIALS AND METHODS

### Clinical specimens

Blocks of paraffin-embedded, formalin-fixed human larynx, obtained from surgical resection specimens, were obtained in accordance with Local Research Ethics Committee guidelines.

The tissues examined represented normal laryngeal squamous epithelium taken from excision margins distant from a resected tumour (*n*=10), LD (*n*=20) and laryngeal SCC (*n*=10). The 20 dysplastic lesions were graded according to the Ljubljana classification. Of these 20 dysplastic lesions, 10 showed atypical hyperplasia in the Ljubljana classification and 10 represented carcinoma *in situ*. Of the 10 lesions classified as carcinoma *in situ*, five corresponded to LD-3 (WHO) and five represented carcinoma *in situ* in the WHO scheme.

### Primary antibodies

Mouse monoclonal antibodies raised against Mcm-2 (Mukherjee *et al*, 2001; Davies *et al*, 2002), Mcm-5 (Mukherjee *et al*, 2001) and Ki67 (Mib-1 clone, DAKO, Ely, UK) were used.

### Immunohistochemistry

Sections (5 *μ*m) were cut onto aminopropyltriethoxysilane (APES)-coated slides and processed for immunohistochemistry, as described previously (Freeman *et al*, 1999).

Primary antibody (100 *μ*l) was applied in a humidified chamber at 4°C overnight with gentle shaking in 1% BSA/TBS with 0.1% Triton X-100. The slides were then washed in TBS containing 0.025% Triton X-100 and incubated for 1 h with biotinylated goat anti-mouse secondary antibody (DAKO, Ely, UK). A streptavidin–horseradish peroxidase system (DAKO, Ely, UK) with the substrate diaminobenzidine was used to develop the stain. The slides were then lightly counterstained with Harris' haematoxylin, dehydrated in increasing concentrations of alcohol and cleared in xylene. Coverslips were applied with DEPEX mounting medium (Gurr, BDH, Poole, Dorset, UK).

Negative controls were performed, for all tissues studied, by omitting the primary antibody. Sections of cervix showing various grades of cervical intraepithelial neoplasia were used as positive controls (Freeman *et al*, 1999).

### Double labelling immunofluorescence for confocal microscopy

In order to examine the differential expression of Mcm-2 and Ki67 in individual specimens, double labelling immunofluorescence experiments were performed on sections of all grades of epithelium (*n*=6).

The sections were prepared as described above. No quenching of endogenous peroxidase was necessary. The Mcm-2 antibody was applied first and, after washing, the sections were incubated with Alexa Fluro goat anti-mouse 488 (Molecular Probes, Eugene, OR, USA). This was followed by a blocking step with F(ab)_2_ goat anti-mouse IgG fragments (Jackson Immuno Research Laboratories, West Grove, PA, USA). A further washing step was then performed before incubation with the Ki67 antibody. Following a final washing step and incubation in Alexa Fluro goat anti-mouse 546 (Molecular Probes), the slides were counterstained and mounted as described above. As an internal control, the order of application of the primary antibodies was reversed.

Images were viewed and assessed using a Zeiss Axioplan 2 confocal microscope at wavelengths of 488 and 546 nm.

### Quantification of antibody staining

For each marker, a semiquantitative indication of staining frequency was determined by calculating a labelling index (LI). In the normal larynx and in lesions showing epithelial hyperplasia or carcinoma *in situ*, the epithelium was divided into equal thirds. The ratio of immunopositive to total epithelial cells was determined for the entire epithelium and for four epithelial compartments, the superficial, middle and lower thirds of the epithelium and the basal layer. In the SCCs, where epithelial orientation is lost, the LI was determined as a mean of five individual counts. The counts were repeated independently by four observers (PC, ISS, LSM, RJD), and an interobserver variation of less than 5% was seen. The overall LI values used represented a mean of the LIs from the four observers.

### Immunocytochemical analysis of laryngeal brushings

The cytological study was performed with the approval of the Cambridge, Harold Wood and Royal Free Hospital Local Research Ethics Committees. Patients undergoing laryngeal biopsy under general anaesthesia for clinically suspicious lesions of the larynx were first submitted to brushing of the mucosa overlying the lesion using a Micro-invasive Brush (Boston Scientific, Watertown, MA, USA). This was applied firmly but without undue pressure for a period of 5 s, following which the tip was cleanly amputated using wire cutters and dropped into PreservCyt® solution (Cytyc Corporation, Boxborough, MA, USA). The solution was then filtered through a TransCyt® filter and the cellular material deposited on a slide in a ThinPrep 2000® cytoprocessor according to the manufacturer's protocols (Cytyc Corporation).

Immunocytochemical staining of the slides was performed on a DAKO Universal Stainer (DAKO, Ely, Cambs, UK). The slides were treated with 4 mM sodium deoxycholate and washed in TBS. A combination of Mcm-2 and Mcm-5 primary antibodies was used and a DAKO Chem Mate™ HRP-detection kit was employed for all subsequent steps. The slides were counterstained with a modified Papanicolaou method (Williams *et al*, 1998) on a Leica Autostainer XL (Leica, Houston, TX, USA) and were then mounted in DPX.

Slides were evaluated by two observers (ISS, LSM), who were blind to the histological diagnosis made in the biopsy. Slides were assessed for Mcm-2/5 immunopositivity and for any atypia demonstrated by the modified Papanicolaou counterstain. The accompanying biopsies were stained with haematoxylin and eosin and also by immunohistochemistry for expression of Mcm-2.

### Statistical analysis

Differences between Mcm-2 and Ki67 LIs were compared using the Bland and Altman limits of agreement analysis. Labelling indicies were compared using the Friedman test, and pairwise comparisons between normal larynx and dysplasia were made using the Mann–Whitney *U*-test. Differences in LIs in the progression from normal larynx through the various grades of hyperplasia and carcinoma *in situ* to SCC were assessed using the Jonckheere–Terpstra test. For initial comparisons, the hyperplastic and carcinoma *in situ* subdivisions were analysed together to give greater statistical power.

## RESULTS

### Immunohistochemical staining

**Figure 1:**
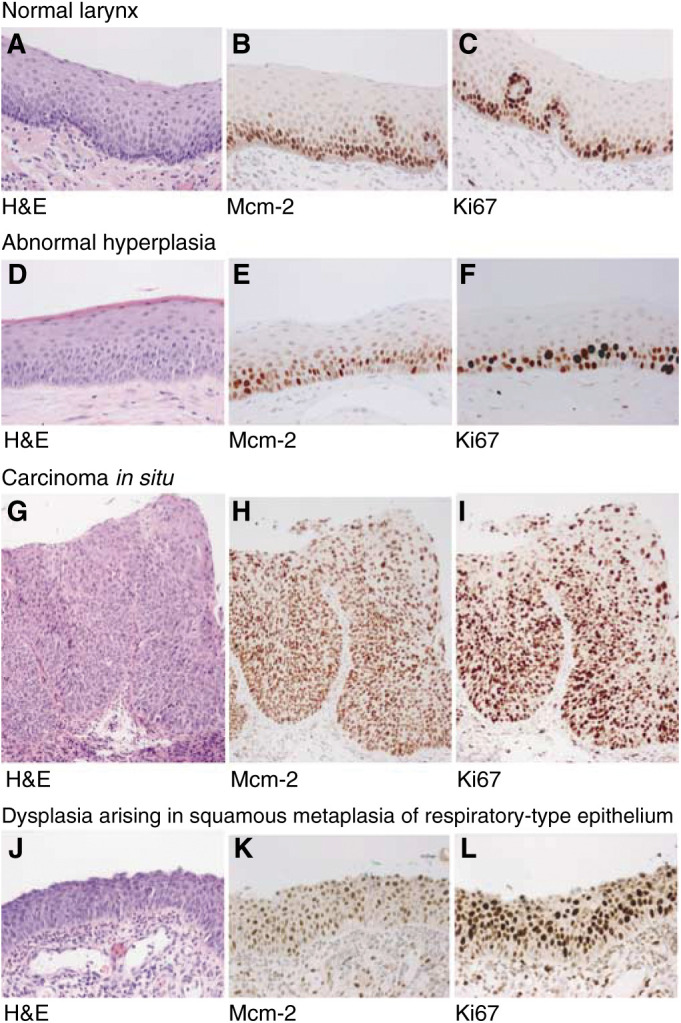
Immunohistochemical staining showing the distribution of Mcm-2 (middle panel) and Ki67 (right panel) in normal laryngeal squamous epithelium, abnormal hyperplasia, carcinoma *in situ* and dysplasia arising in squamous metaplasia affecting respiratory-type epithelium. The haematoxylin & eosin (H&E) appearances are given for comparison (left panels). All figures are at × 200 magnification. In carcinoma *in situ*, there is full-thickness staining for both markers, with clusters of immunopositive cells that appear to have sloughed away from the epithelial surface.

Sections of normal laryngeal epithelium showed a normal pattern of maturation from the basal layer to the surface (Figure 1A

). Very little surface keratin was seen in the absence of inflammation. Expression of Mcm-2 and Ki67 was detected in occasional cells within the basal layer (Figure 1B and C). There was expression of Mcm-2 in approximately 60% of cells in the lower third of the epithelium. Very few cells were positive in the middle third and there was no staining with either antibody in the superficial third.

In lesions showing abnormal or atypical hyperplasia (Figure 1D), Mcm-2 and Ki67 were expressed in the lower and middle thirds, with only occasional cells being positive in the surface layers (Figure 1E and F).

In lesions showing carcinoma *in situ* (WHO: LD-3, CIS), dysplastic cells occupy the lower and middle thirds and extend into the superficial third of the epithelium (Figure 1G). In these high-grade lesions, Mcm-2 and Ki67 immunopositive cells were abundant in all three layers, with frequent extension to the epithelial surface (Figure 1H and I). In addition, occasional cells showing Mcm-2 and Ki67 immunoreactivity appeared to have sloughed away from the epithelial surface (Figure 1H and I).

We also observed areas of dysplasia in squamous metaplasia arising in regions originally showing a respiratory-type (pseudostratified) columnar epithelium (Figure 1J). These areas also showed full-thickness expression of both Mcm-2 and Ki67 (Figure 1K and L).

In SCC, there was widespread expression of both Mcm-2 and Ki67, with mean LIs of 80.6 and 63.9%, respectively.

### Mcm-2 and Ki67 LIs in different epithelial compartments

**Figure 2:**
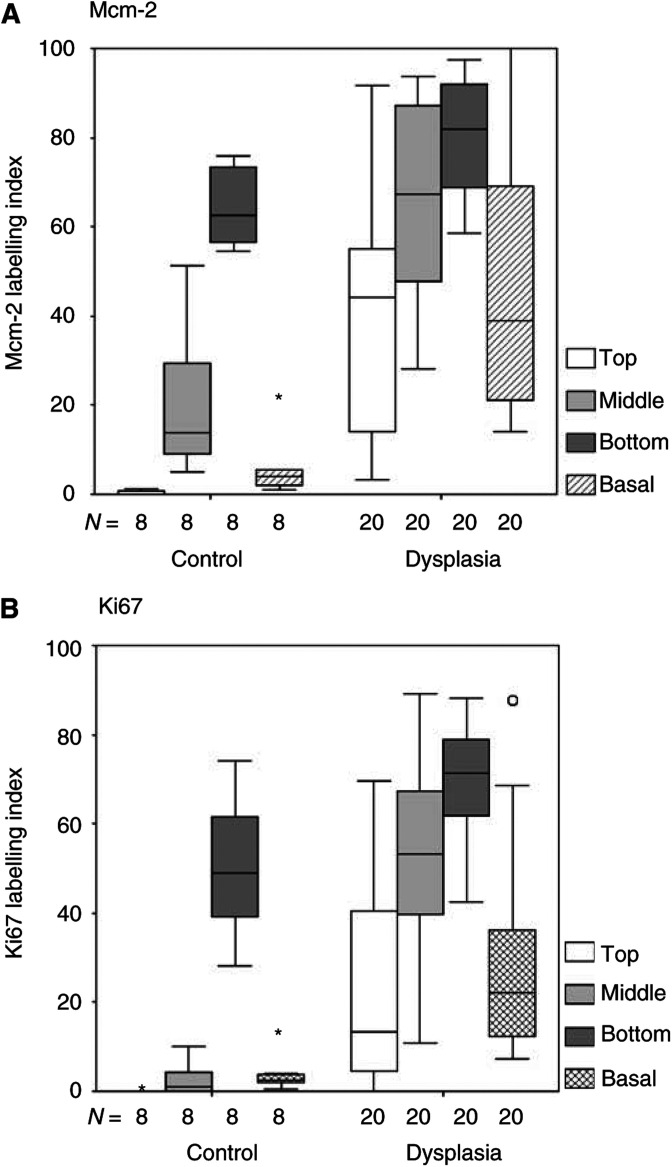
Box and whisker plots comparing Mcm-2 (**A**) and Ki67 (**B**) LIs in different compartments of normal laryngeal squamous epithelium and LD (atypical hyperplasia and carcinoma *in situ* combined). Control=normal larynx, bar=median value, box=upper to lower quartile ranges, whisker=2 × quartile range, asterisk and circle=extreme values.

In normal larynx and in lesions showing dysplasia (atypical hyperplasia and carcinoma *in situ* combined), Mcm-2 expression decreased from the lower to the middle third and from the middle to the superficial third (Figure 2A

) (*P*<0.0001). Mcm-2 expression in the basal layer was low, and was similar to that seen at the surface. There was no overlap in Mcm-2 LI values between the four compartments of normal epithelia analysed, although overlap was seen for dysplastic lesions (Figure 2A).

Ki67 LIs showed similar patterns to those of Mcm-2 (Figure 2B). In normal larynx, an increase in expression in the middle third of the epithelium compared to the superficial third was identified, although the LI values at these levels did overlap. There was a sharp increase in Ki67 LI in the lower third of the epithelium compared to the middle third, with no overlap in LI values. The basal layer showed a low LI, as seen with Mcm-2. Ki67 LIs in the various compartments of dysplastic epithelia were also similar to those for Mcm-2 (Figure 2B). Overall, as with Mcm-2, in normal larynx and laryngeal dysplasia there was a highly significant difference in Ki67 LI values between the four epithelial compartments studied (*P*<0.0001).

### Mcm-2 and Ki67 expression in atypical hyperplasia/carcinoma *in situ vs* normal epithelia

**Figure 3:**
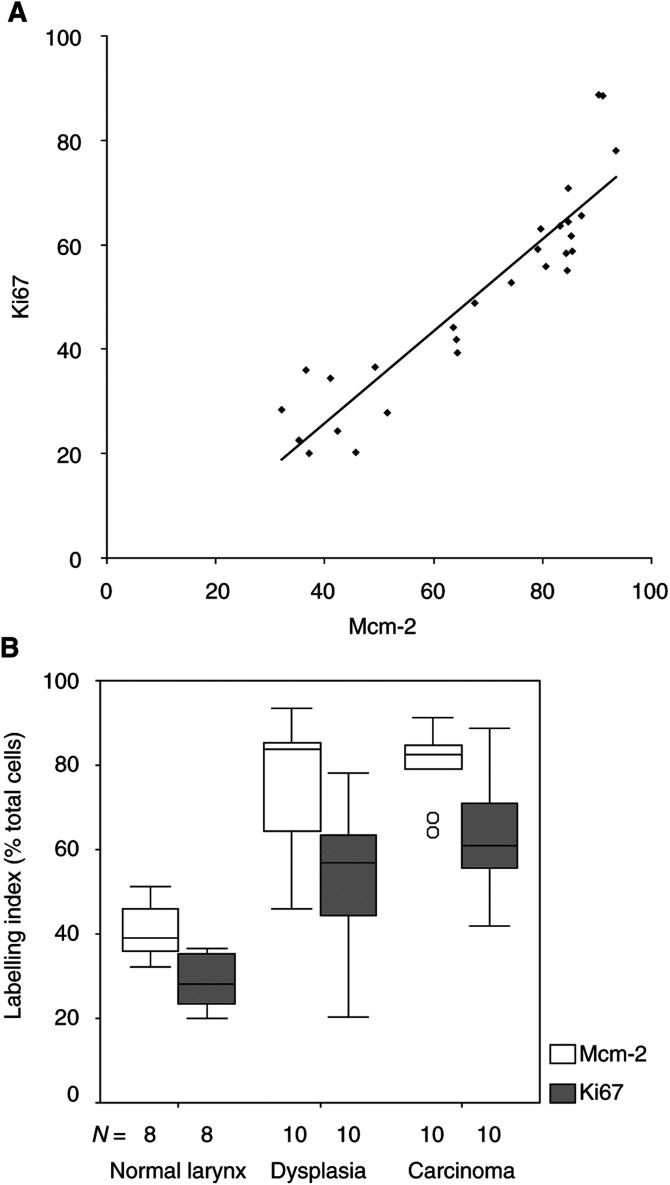
(**A**): Correlation between Mcm-2 and Ki67 LIs for the entire epithelium in normal larynx (*n*=10), LD unaccompanied by invasive disease (*n*=10) and SCC (*n*=10) (*ρ*=0.93; 95% CI [0.84, 0.97]). (**B**) Comparison between Mcm-2 and Ki67 LIs for the entire epithelium in normal larynx (*n*=8), LD unaccompanied by invasive disease (*n*=10) and laryngeal SCC (*n*=10; median±IQR). Box=upper to lower quartile ranges; whisker=2 × quartile range; circles=extreme values.

Overall, there was a significant correlation between Mcm-2 and Ki67 LIs (*ρ*=0.93; 95% CI [0.84, 0.97]), although Mcm-2 expression was elevated when compared to Ki67 (Figure 3A

). There was an increase in median LIs for the entire epithelium of both Mcm-2 and Ki67 in the progression from normal larynx through dysplasia to SCC (Mcm-2, *P*=0.001; Ki67, *P*=0.0002) (Figure 3B).

**Figure 4:**
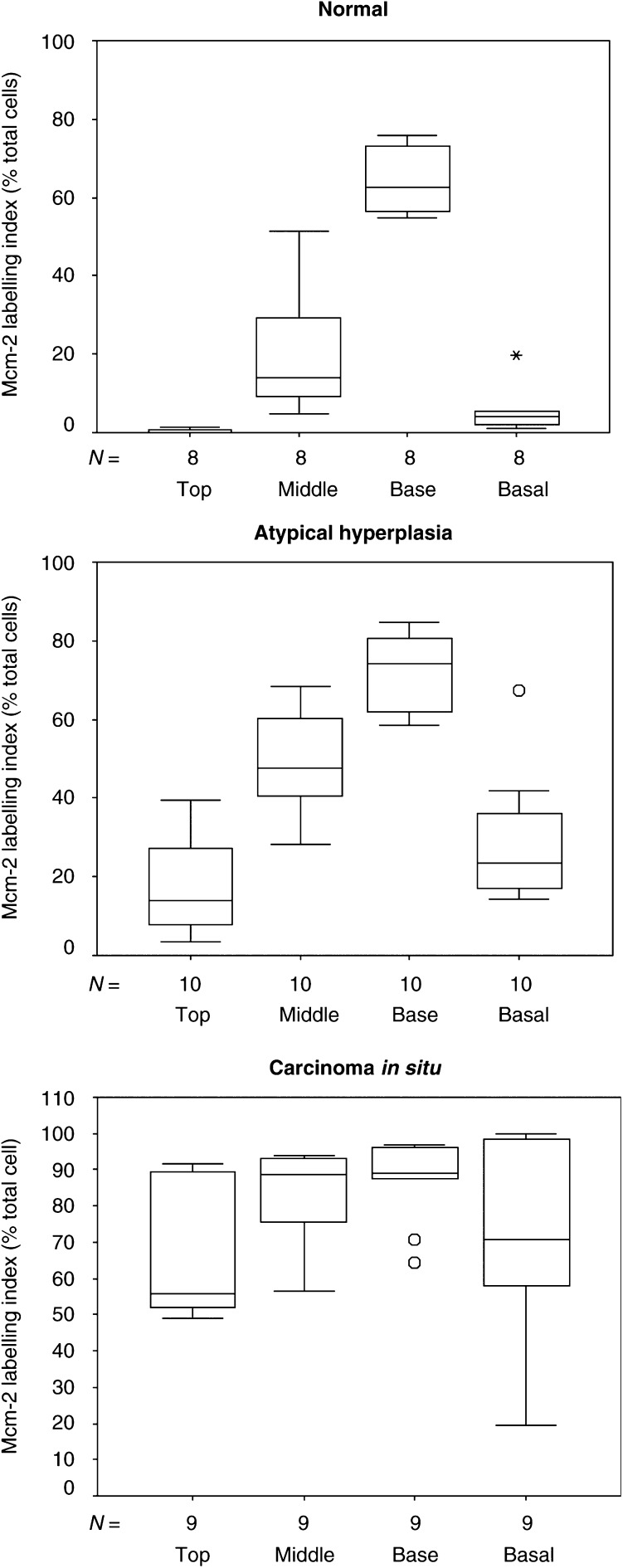
Bar charts (median±IQR) comparing Mcm-2 LIs in different compartments of the laryngeal epithelium in normal larynx (*n*=8), atypical hyperplasia (*n*=10), carcinoma *in situ* (*n*=9). Substantial expression of Mcm-2 in the superficial epithelial third is only present in carcinoma *in situ*, although some increase in expression above normal is seen in atypical hyperplasia. Base=lower epithelial third, asterisk and circle=extreme values.

Mcm-2 LIs were significantly greater in lesions showing dysplasia (atypical hyperplasia and carcinoma *in situ* combined) in comparison to normal larynx in the superficial (*P*<0.0001), middle (*P*<0.0001) and lower thirds of the epithelium (*P*=0.003) and in the basal layer (*P*<0.0001) (Figure 2A). Substantial expression of Mcm-2 in the superficial third was only seen in carcinoma *in situ* (WHO: LD-3, CIS), although some increase in expression was seen in atypical hyperplasia (WHO: LD-1/2) (Figure 4

).

Ki67 LIs showed similar differences, with greater values in the upper (*P*<0.0001), middle (*P*<0.0001) and lower (*P*=0.008) thirds and in the basal layer (*P*<0.0001) of dysplastic lesions when compared to normal larynx (Figure 2B).

### Confocal microscopy

**Figure 5:**
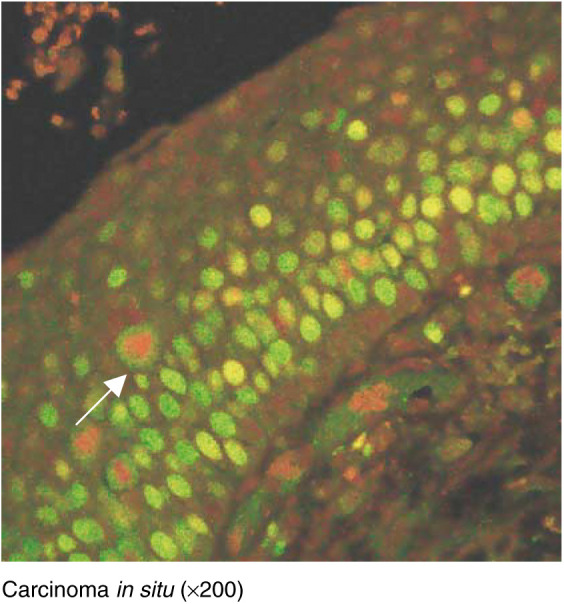
Double-labelling fluorescent confocal microscopy for Mcm-2 (green) and Ki67 (red) in carcinoma *in situ*. Many cells coexpress Mcm-2 and Ki67 (yellow), but there are also many cells showing Mcm-2-expression alone (green). Mitotic figures show chromatin exclusion of Mcm-2 (red staining) but the cytoplasm is positive for Mcm-2 (arrow). Some Mcm-2-expressing cells (green) are present in the superficial third whereas very few cells express Ki67 (yellow).

In view of the greater frequency of expression of Mcm-2 compared to Ki67 (Figures 2 and 3), we compared expression of the two molecules in individual specimens using double-labelling confocal fluorescence microscopy (Figure 5

). In all cases, all cells expressing Ki67 (red) also expressed Mcm-2 (green), giving a yellow signal. No cells showing a red fluorescent signal (Ki67 alone) were identified, indicating that Mcm-2 was present in all cells expressing Ki67. There were, however, many cells expressing Mcm-2 (green) that did not express Ki67 (red), in all grades of epithelium.

### Laryngeal brushings

**Figure 6:**
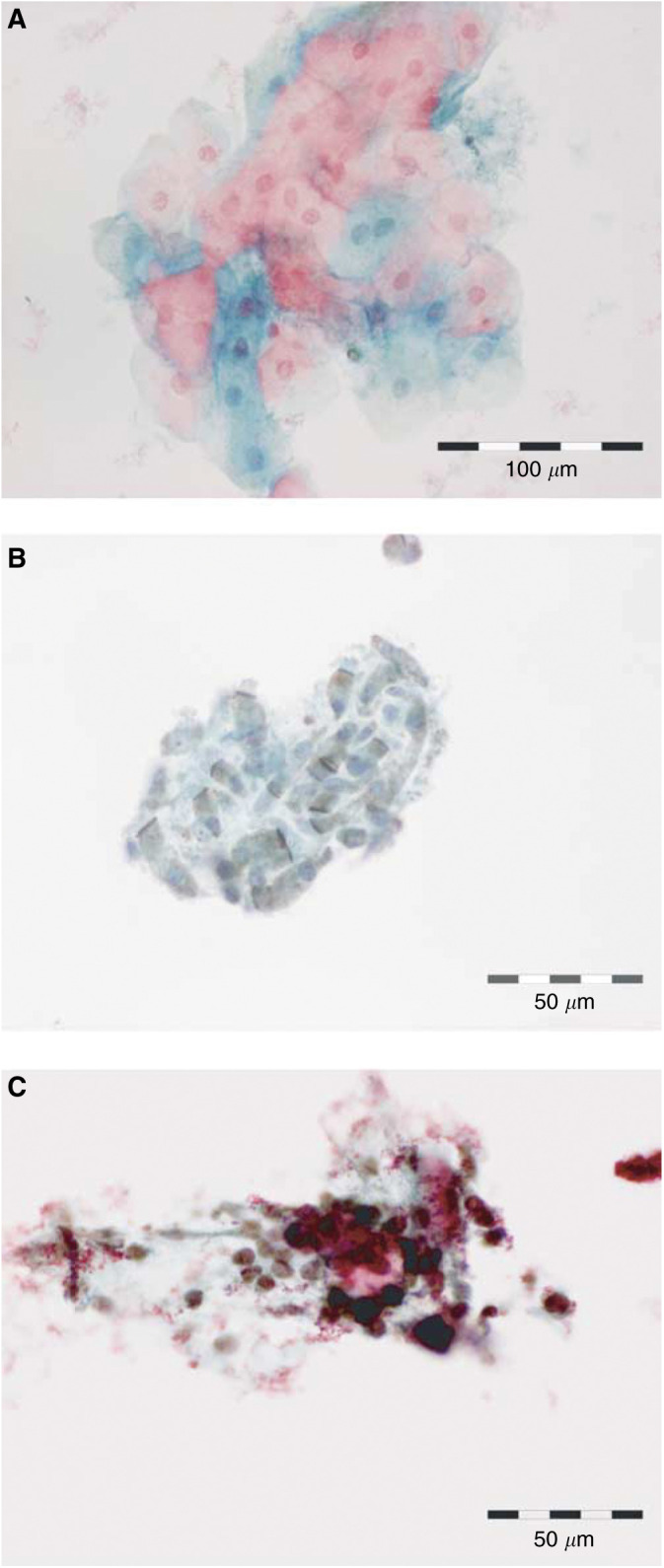
Normal squamous (**A**) and respiratory-type (**B**) epithelial cells are negative when stained with antibodies against Mcm-2/5. (**C**) A cluster of cells from a laryngeal SCC, which are strongly positive for Mcm-2/5 and were readily identified, even at low magnification.

In view of the high frequency of expression of Mcm-2 in the superficial third of dysplastic epithelia (including in sloughed cells), we undertook a small pilot study to investigate whether laryngeal lesions could be detected by immunocytochemical analysis of MCM proteins in laryngeal brushings. Brushings were obtained from lesions (*n*=6) from which a biopsy was also taken as a gold standard (Figure 6

).

In two cases, histological examination showed parakeratosis and inflammation only, with Mcm-2 staining only in the basal layer of the epithelium. Brushings taken from these specimens showed no cytological atypia and no evidence of Mcm-2/5 immunopositivity.

A third biopsy revealed the presence of atypical hyperplasia (WHO: LD-1/2), with parakeratosis and koilocytosis indicating HPV infection. Minichromosome maintenance protein-2 staining extended into the middle third of the epithelium, but there was no Mcm-2 expression at the epithelial surface. Cytological brushings from this case showed mild cytological atypia, but there was no Mcm-2/5 immunopositivity.

The remaining three biopsies all showed invasive SCC. In all cases, immunohistochemistry for Mcm-2 showed widespread and frequent staining of malignant cells. Brushings from these cases all showed moderate to severe cytological atypia and the majority of atypical cells expressed Mcm-2. Immunopositive cells stood out clearly in the cytological preparations and were readily identified, even at low magnification.

## DISCUSSION

Recent epidemiological findings suggest that there are considerable differences in the aetiology of cervical and laryngeal cancer. A link between HPV infection and cervical cancer has been demonstrated in over 99% of specimens (Walboomers *et al*, 1999). Recent investigations, on the other hand, do not support HPV as an important factor in the pathogenesis of laryngeal epithelial lesions (Brandwein *et al*, 1993), although the prevalence of HPV in laryngeal carcinomas has been found to range from 5 to 54.1% (Hoshikawa *et al*, 1990; Perez-Ayala *et al*, 1990; Brandwein *et al*, 1993). The aetiology of laryngeal cancer is more likely to be related to cigarette smoking, alcohol abuse and exposure to other extrinsic irritants. These probably trigger a series of genetic events that differ from those in the cervix (Michaels, 1992). It would be expected, therefore, that the histological features of precancerous lesions of the larynx would be different from those seen in the cervix, and this is indeed the case. The Ljubljana classification is designed to take into account these differences and to predict more accurately the biological behaviour of precancerous lesions of the larynx. This is important as repeat smears/biopsies cannot readily be performed in the larynx and there is a pressing need to differentiate accurately between potentially malignant and actual preinvasive carcinoma as these two lesions are treated differently at this site (Hellquist *et al*, 1999).

Our comparison of the expression of Mcm-2 with that of Ki67, an established marker of cell cycle entry, shows a direct correlation between the frequency of expression of the two proteins. However, Mcm-2 is detectable in greater numbers of cells at the surface of premalignant laryngeal lesions. This observation is supported by our double-labelling confocal microscopy data showing that Mcm-2 is expressed not only in all Ki67-positive cells but also in Ki67-negative cells.

Our histological data show that Mcm-2 is expressed within the most superficial surface layers of laryngeal epithelium in cases of carcinoma *in situ* and SCC, with minimal expression in abnormal and atypical hyperplasia. We suggest, therefore, that Mcm-2 would be a good candidate as a biomarker of premalignant and malignant laryngeal disease. It has been suggested that the Ljubljana classification can be made more accurate with the silver-staining of nucleolar organising regions, or using Ki67 (Grundmann, 1983). Our data show that Mcm-2 is superior to Ki67 in the detection of cell cycle entry in the larynx. Application of Mcm-2 immunohistochemistry could, therefore, increase the diagnostic accuracy of the Ljubljana classification, and further work in this area is warranted.

In the cervix, a comparable squamous epithelium to that of the larynx, routine cytological surveillance has been shown to be highly effective in identifying premalignant disease (Vizcaino *et al*, 2000). Cervical screening requires removal of surface epithelial cells by scraping or brushing. Any malignant or potentially premalignant cells so sampled can be identified using morphological or immunocytochemical parameters. We have previously shown that immunocytochemistry for Mcm-2 in cervical smears enables abnormal cells to be identified with very high sensitivity (Williams *et al*, 1998). Based on our immunohistochemical data in the present study, we reasoned that, by analogy with our findings in the cervix, if the superficial cells in the larynx are removed by brushing, Mcm-2 expression should be detected in cytological preparations obtained from malignant or premalignant lesions. Importantly, such an approach would enable all of the laryngeal epithelium to be sampled. We observed that in carcinoma *in situ* and SCC, many Mcm-2-positive cells appear to be sloughed from the epithelial surface and this should assist in the detection of these lesions by laryngeal brushing. Indeed, our small pilot study suggests that Mcm-2/5 immunocytochemistry combined with liquid-based cytology may enable laryngeal SCC to be distinguished from hyperplastic lesions and non-neoplastic epithelium following laryngeal brushing.

Our experience to date suggests that laryngeal brushing is a technique that can be performed under general anaesthesia, although ultimately it would be preferable if the technique could be adapted for use in the outpatient setting under topical anaesthesia. The method causes little discomfort, is relatively cost efficient and could provide a means of assessing large numbers of patients with early nonspecific symptoms. Based on the results from liquid-based cytology enhanced by immunocytochemistry for Mcm-2/5, these patients could be divided into those requiring further investigation and those who could be followed up without resort to biopsy.

There is, therefore, the potential for laryngeal brushing to be developed further in the context of less-invasive detection of early laryngeal epithelial lesions, although this will ultimately be dependent upon the availability of flexible nasoendoscopic equipment and refinement of techniques to allow the use of a fine brush as an outpatient procedure.
